# Ectopic pregnancy incidence in the Republic of Korea in 2009–2015: A population-based cross-sectional study

**DOI:** 10.1038/s41598-018-35466-5

**Published:** 2018-11-23

**Authors:** Ji Eun Park, Jin-Sung Yuk, In Ae Cho, Jong Chul Baek, Jung-hun Lee, Ji Kwon Park

**Affiliations:** 1 Department of Obstetrics and Gynecology, College of Medicine, Gyeongsang National University, Gyeongsang National University Changwon Hospital, Changwon, 51472 Republic of Korea; 2 Department of Obstetrics and Gynecology, College of Medicine, Eulji University, Nowon Eulji Medical Center, 68, Hangeulbiseok-ro, Nowon-gu, Seoul, 01830 Republic of Korea; 3 Department of Obstetrics and Gynecology, College of Medicine, Gyeongsang National University, Gyeongsang National University Hospital, Jinju, 52727 Republic of Korea

## Abstract

We estimated the incidence of ectopic pregnancy (EP) and the success rate of expectant management of EP in South Korea. We analyzed data from 2009 to 2015 using the Health Insurance Review and Assessment Service National Inpatient Sample (HIRA-NIS) database. EP was identified by diagnostic codes, and strict EP was identified by both diagnostic codes and treatment codes. From 2009 to 2015, 369,701 cases of EP, abortion, or delivery were extracted from a total of 4,476,495 women. Of the total pregnancies, 8,556 cases were EPs. The incidence of EP was 34.1 ± 0.7 per 1,000 pregnancies and the incidence of strict EP was 17.3 ± 0.3 per 1,000 pregnancies. Among women aged 25–44 years, age was associated with a higher incidence of EP (odds ratio [OR]: 1.13; 95% confidence interval [CI]: 1.06, 1.19; *P* < 0.01). The incidence rates of EP (OR: 0.99; 95% CI: 0.97, 1.01; *P* = 0.51) did not significantly differ by year. The incidence of EP in Korea was 17.3 ± 0.3 per 1,000 pregnancies, and almost did not change over 7 years. About 50% of EPs were treated without surgery or methotrexate. This study provides an important reference for the treatment of EP.

## Introduction

Ectopic pregnancy which occurs in 2% of all spontaneous conceptions, is the most common cause of mortality during the first trimester of pregnancy and is responsible for up to 7% of all pregnancy-related deaths^1–4^. Treatment options include surgery through laparoscopy or laparotomy, medical therapy with methotrexate, or expectant management. The cost of treating an EP is significant; direct costs are estimated at $1 billion in the US^5^. EP is of medical importance because it is associated with reduced fertility and increased risk of subsequent EP^6^. Thus, it is clinically important to monitor the incidence of EP.

The overall incidence of EP was 19.7 per 1,000 pregnancies in 1992, the last time national data were reported by the U.S. Centers for Disease Control and Prevention^4^. Since then, its incidence has been reported by private insurers and local health care providers. Van Den Eeden *et al*. reported a rate of 20.7 per 1,000 pregnancies in the Kaiser Permanente Northern California region from 1997–2000^7^. Trabert *et al*. reported that in patients in the Group Health Cooperative health care plan for Washington and Idaho, the incidence increased from 17.8 per 1,000 pregnancies in 1993–1995 to 21.1 per 1,000 pregnancies in 2005–2007^8^. Hoover *et al*. reported a treatment rate of EPs of 6.4 per 1,000 pregnancies from 2002–2007 among women in MarketScan, a database of US commercial insurers^9^. The prevalence rate of EP in England and Wales in 1994–1996 was reported as 12.4 per 1,000 pregnancies, and the incidence in French women in 1992 was reported as 15.8 per 1,000 pregnancies. The frequency of EP in the Republic of Korea was estimated as 15.8 per 1,000 pregnancies in 2009^10–12^. However, the data from these studies are not recent, and the study populations were relatively small. Therefore, new estimates are needed.

Typical treatment options for EP include surgery, such as laparoscopy and drug therapy with methotrexate. However, expectant management is considered for some visible EPs, in cases of pregnancy of unknown location (PUL) when there are low or plateauing serum human chorionic gonadotropin (hCG) concentrations^13,14^. Expectant management is based on the knowledge that the natural history of many early EPs is a self-limiting process that ultimately results in tubal abortion or reabsorption^15,16^. Improved diagnostic methods, such as more sensitive pregnancy tests and high-grade vaginal ultrasonography, have made it possible to diagnosis EP early, including PUL, before the patient’s condition has deteriorated. With timely diagnosis, clinicians can consider all treatment options; this has increased the number of laparoscopic surgeries and the frequency of medical management with methotrexate^9^. However, few population-based studies have assessed the success rates of expectant management in EP.

We estimated the incidence of EP in Korea from 2009 to 2015, and the success rate of expectant management of EP using the Korean Health Insurance Review and Assessment Service National Inpatient Sample (HIRA-NIS) database.

## Materials and Methods

### Study Settings and Participants

The National Health Insurance Corporation (NHIC) provides medical services to more than 98% of the South Korean population, which is approximately 49 million^17^. We performed stratified random sampling (1 million) of all South Koreans by year in the HIRA-NIS database. The data were extracted using a weighted sampling method, which extracted 13% from the inpatient population and 1% from the outpatient population^17^.

We analyzed HIRA-NIS data from 2009 to 2015 (serial numbers: HIRA-NIS-2009-0066, HIRA-NIS-2010-0084, HIRA-NIS-2011-0063, HIRA-NIS-2012-0058, HIRA-NIS-2013-0085, HIRA-NIS-2014-0068, and HIRA-NIS-2015-0057). We used the Korean Standard Classification of Diseases (KCD), fifth revision, HIRA Drug Ingredient codes, and Health Insurance Medical Care Expenses, 2016 version, to calculate the number of pregnancies.

EP was defined by the diagnostic code O00 (EP), and abortion was defined by the diagnostic codes O02 (other abnormal products of conception), O03 (spontaneous abortion), O04 (medical abortion), O05 (other abortion), and O06 (unspecified abortion). In this study, abortion was defined as early pregnancy loss, regardless of whether it was a spontaneous or induced event. Delivery was defined by the diagnostic codes O80 (delivery in a completely normal case), O81 (single delivery by forceps and vacuum extractor), O82 (single delivery by cesarean section), O83 (other assisted single delivery), and O84 (multiple delivery). Antenatal care of normal pregnancy was defined by the diagnostic codes Z33 (pregnant state, incidental) and Z34 (supervision of normal pregnancy). Because diagnoses of EP, abortion, and antenatal care of normal pregnancy may be exclusionary rather than confirmed diagnoses, the final diagnostic code among all consecutive diagnostic codes applied within 60 days is defined as the final diagnosis. For example, if there was a diagnostic code for an EP but also a diagnostic code for antenatal care of normal pregnancy 30 days later, the case was defined as an antenatal care case rather than an EP case. If there was a diagnostic code for an EP, abortion, or antenatal care of normal pregnancy at an interval of more than 60 days, it was considered as a separate episode. The total number of pregnancies included delivery, EP, and abortion.

Antenatal care cases were excluded from the total number of pregnancies to avoid duplicate calculations. Strict EP was defined as having an EP diagnostic code while also having an EP operation code–R4531 (tubal or ovarian pregnancy), R4532 (cornual pregnancy), R4533 (cervical pregnancy), or R4534 (abdominal pregnancy) –or the methotrexate drug code (19210XXX). The incidence of EP was calculated by dividing the total number of episodes of EP by the sum of the episodes of EP, abortion, and delivery. The incidence of strict EP was calculated by dividing the total number of episodes of strict EP by the sum of the total number of episodes of EP, abortion, and labor.

### Statistical analysis

All statistical analyses were performed with R software (ver. 3.3.2; R Foundation for Statistical Computing, Vienna, Austria). A *P* value < 0.05 was considered statistically significant. All statistical tests were two-sided. Weighted analyses were used to compare means of continuous variables, and the weighted Pearson’s chi-square test was used for statistical analyses of categorical variables.

### Ethics

This study was not subject to review by the Institutional Review Board in accordance with the Korean Bioethics and Safety Act, because it used existing data that did not contain any personally identifiable information.

## Results

**Figure 1:**
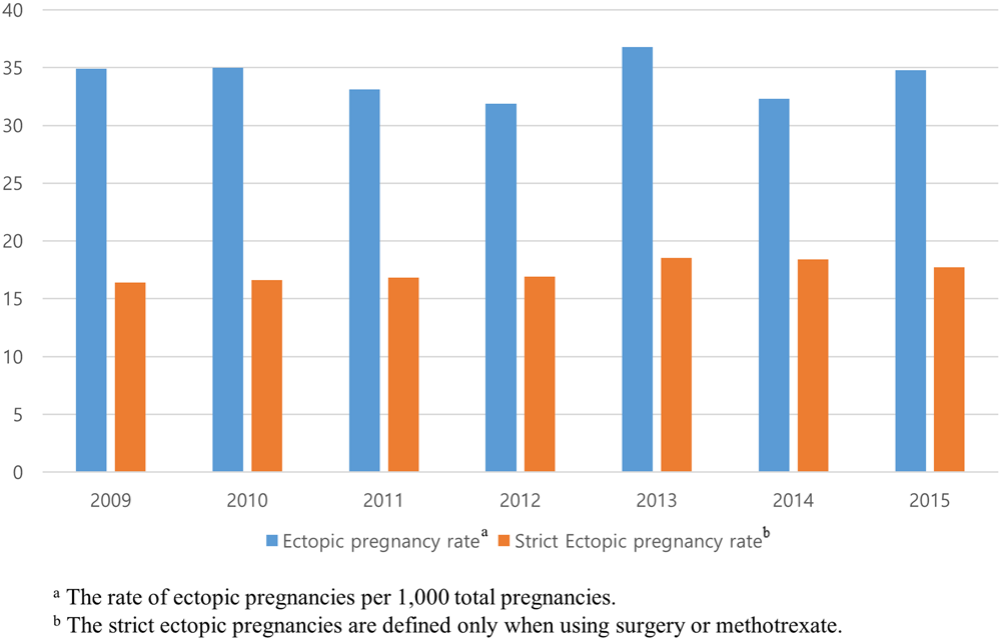
Trend of ectopic pregnancy (EP) incidence from 2009 to 2015. The rate of ectopic pregnancies per1,000 total pregnancies. Strict ectopic pregnancies are defined only when using surgery or methotrexate.

**Figure 2:**
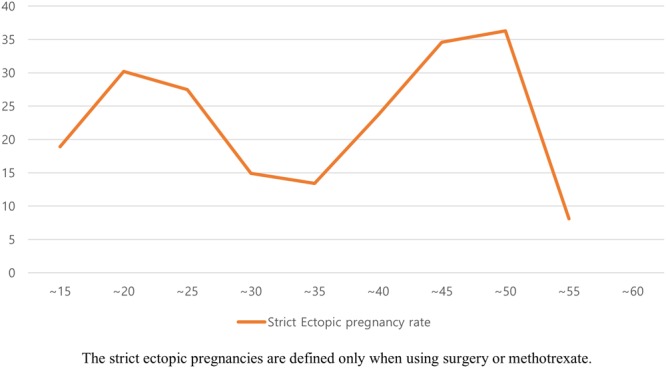
Trend of EP incidence by age. Strict ectopic pregnancies are defined only when using surgery or methotrexate.

From 2009 to 2015, 369,701 cases of EP, abortion, or delivery were extracted from a total of 4,476,495 women. Of the total pregnancies, 8,556 cases were EPs and 361,145 cases were non-EPs. The mean ages were 31.1 ± 0.0 years and 31.9 ± 0.1 years, respectively (*P* < 0.01). The composition of each pregnancy case by calendar year is shown in Table 1. The incidence of EP was 34.1 ± 0.7 per 1,000 pregnancies and the incidence of strict EP was 17.3 ± 0.3 per 1,000 pregnancies (Table 2). EPs requiring surgery or methotrexate treatment made up 50.7 ± 1.1% of total EPs. Figures 1 and 2 show the incidence of EP by year and age, respectively. According to weighted logistic regression analyses adjusted for year and age (per 5 years), the incidence of EP (odds ratio [OR]: 1.13; 95% confidence interval [CI]: 1.06, 1.19; *P* < 0.01) and the incidence of strict EP (OR: 1.12; 95% CI: 1.07, 1.17; *P* < 0.01) increased with age but did not change by year (EP OR: 0.99; 95% CI: 0.97,1.01; *P* = 0.51; strict EP OR: 1.01; 95% CI: 1.00, 1.03; *P* = 0.13). The distribution of the sites of EP by surgical operation was as follows: 91.5% tubal or ovarian pregnancy, 5.9% corneal pregnancy, 1.9% cervical pregnancy, and 0.9% abdominal pregnancy.

**Table 1:** Each pregnancy cases according to the year from 2009 to 2015 according to HIRA-NIS.

	2009	2010	2011	2012	2013	2014	2015	p
	(N = 53,195)	(N = 56,566)	(N = 56,721)	(N = 55,588)	(N = 49,451)	(N = 49,106)	(N = 49,074)	
Age (y)	30.2 ± 4.3	30.6 ± 11.6	30.8 ± 14.7	30.9 ± 4.1	31.2 ± 4.1	31.2 ± 4.3	31.3 ± 4.3	0
Ectopic pregnancy								0.007
0	51,851 (97.5%)	55,213 (97.6%)	55,411 (97.7%)	54,397 (97.9%)	48,304 (97.7%)	47,976 (97.7%)	47,993 (97.8%)	
1	1342 (2.5%)	1353 (2.4%)	1309 (2.3%)	1189 (2.1%)	1147 (2.3%)	1129 (2.3%)	1081 (2.2%)	
2	2 (0.0%)	0 (0.0%)	1 (0.0%)	2 (0.0%)	0 (0.0%)	1 (0.0%)	0 (0.0%)	
Abortion								0
0	50,176 (94.3%)	53,279 (94.2%)	53,600 (94.5%)	52,690 (94.8%)	46,556 (94.1%)	45,961 (93.6%)	46,125 (94.0%)	
1	3018 (5.7%)	3285 (5.8%)	3121 (5.5%)	2897 (5.2%)	2894 (5.9%)	3145 (6.4%)	2949 (6.0%)	
2	1 (0.0%)	1 (0.0%)	0 (0.0%)	1 (0.0%)	1 (0.0%)	0 (0.0%)	0 (0.0%)	
3	0 (0.0%)	1 (0.0%)	0 (0.0%)	0 (0.0%)	0 (0.0%)	0 (0.0%)	0 (0.0%)	
Delivery								0
0	4269 (8.0%)	4519 (8.0%)	4335 (7.6%)	4014 (7.2%)	3967 (8.0%)	4212 (8.6%)	3957 (8.1%)	
1	48,926 (92.0%)	52,047 (92.0%)	52,385 (92.4%)	51,574 (92.8%)	45,484 (92.0%)	44,894 (91.4%)	45,117 (91.9%)	
2	0 (0.0%)	0 (0.0%)	1 (0.0%)	0 (0.0%)	0 (0.0%)	0 (0.0%)	0 (0.0%)

**Table 2:** Estimated pregnancy rates in 2009~2015 in the Republic of Korea.

	Value ± standard error
Ectopic pregnancy rate^a^	34.1 ± 0.7
Strict ectopic pregnancy rate^a,b^	17.3 ± 0.3
Abortion rate^c^	161.5 ± 1.6
Delivery rate^d^	804.4 ± 1.7

^a^The rate of ectopic pregnancies per 1,000 total pregnancies.

^b^Strict ectopic pregnancies involved the use of surgery or methotrexate.

^c^The rate of abortions per 1,000 total pregnancies.

^d^The rate of deliveries per 1,000 total pregnancies.

## Discussion

The incidence of strict EP was 17.3 ± 0.3 per 1,000 pregnancies, and 50.7% of cases resolved without active treatment.This study is a follow-up of South Korea’s EP rate published in 2013 from HIRA-NIS 2009 data^12^. We updated the EP rate using the newly available HIRA-NIS database from 2009 to 2015. The large national database population used in this analysis provided a sufficient sample size for monitoring the infrequent outcome of EP. It included a sufficient number of insurance claims for EP diagnosis and management to provide the necessary statistical power to accurately measure the rate and trends.

The average annual strict EP rate in our study is similar to the 17.9–20.7 per 1,000 pregnancies reported in other studies^7,8^ but higher than the 6.4 cases per 1,000 pregnancies reported by Hoover *et al*.^9^. However, Hoover *et al*. included both prenatal visits and deliveries in the denominator for calculating EP incidence, whereas other previous studies and our study excluded prenatal visits in the denominator to avoid duplicate calculations. This different methodology appears to have resulted in the much lower incidence reported in that study. In addition to prenatal visits, the addition of elective abortion in the denominator appears to have also affected the incidence rate, although only by a small degree.

In this study, the incidence of strict EP, defined by both diagnostic and treatment codes, was about 50% of the EP incidence calculated using only diagnostic codes. This indicates that about half of the diagnosed EPs were conservatively treated without surgery or methotrexate treatment. This is much higher than reported (20.1%) in a previous study^18^. Improved diagnostic methods such as more sensitive pregnancy tests and high-grade vaginal ultrasonography have made early diagnosis of EP possible, and has likely contributed to the increased use of expectant management in EP. Around 6–20% of women with EP exhibit a continuous decrease hCG levels, and about half of women treated with expectant management are successfully cured^18,19^. On the other hand, in Hoover *et al*., approximately 60% of patients were cured without treatment and showed comparable results to this study^9^. This suggests that the success rate of expectant management is higher in a realistic clinical environment than in a rigorous research environment.

Although advances in diagnostic methods have allowed for earlier diagnosis, EP still remains a life-threatening condition. Approximately 75% of deaths during the first trimester and 9% of all pregnancy-related deaths are due to EP^2^. Thus, surgery is a mainstay of EP management, but the options of medical or expectant management are only available for a subset of patients^15^.

Mathematical models have been developed to predict the development of pregnancies in unknown locations^13^, often using logistic regression analyses. The most widely evaluated model is M4, which incorporates the average hCG concentration measured at 0 and 48 h, the ratio of the two hCG measurements, and the quadratic effects of the hCG ratio^20^. Using this model may help improve the success rate of expectant management of EP. Further research is needed to improve this method.

In calculating the incidence of EP, the denominator includes cases of abortion, EP, and labor. A decrease in artificial abortions should thus increase the total number of abortions, EPs, and deliveries, which should reduce the overall incidence of EPs. However, the rate of strict EP did not change over the 6-year period of our study. This may indicate that there have been no changes in the number of elective abortions in Korea, despite the anti-abortion campaigns that began at the end of 2009^21^. This suggests that civically organized anti-abortion campaigns are ineffective without governmental or political force.

In our study, the rate of EPs among pregnant women tended to increase with age, which is consistent with other studies^9,12^. Several possible explanations may account for this observation. Age-related changes in the structure and function of the fallopian tubes tend to lead to an increased incidence of EP in older women. The risk of gynecological disease such as pelvic inflammatory disease increases with age. Repetitive or asymptomatic episodes of pelvic inflammatory disease may occur over time, which can lead to progressive damage of the fallopian tubes^22^. Assisted reproductive technologies (ARTs) increase the incidence of EP compared to spontaneous conceptions. In addition, more than half of ART procedures are performed in women over 35 years of age^23^. A unique feature of this study is that the rate of EP was higher among women aged 15–25 years than among women aged 25–40 years. This is likely due to socio-cultural factors. Census data from 2014 indicate that the mean age at first marriage was 29.8 years and the mean age at first birth was 30.5 years for women^24^. Younger women were less likely to marry, and more likely to choose elective abortion when pregnant. As a result, the rate of maintaining pregnancy differed depending on age and marital status.

Our study had several limitations. First, elective abortion was not included in the calculation because the Korean National Health Insurance did not cover elective abortion. It is difficult to find cases of elective abortion in a situation where the legal permission for abortion is limited. The number of abortions missed would affect the expected number of total pregnancies. Second, our study did not include pathological or ultrasound records because it used insurance data. Therefore, the accuracy of diagnoses may be relatively low. However, we attempted to limit inaccurate diagnoses by supplementing our classification of EP with the treatment codes for methotrexate or operations.

Nevertheless, we used 7 years of national insurance data to reduce bias and to obtain large-scale cases. Therefore, our analysis will likely provide important information for clinicians seeking to understand the epidemiological characteristics of EP in Korea.

In conclusion, the incidence of EP in Korea was 17.3 ± 0.3 per 1,000 pregnancies, and this rate almost did not change for 7 years. Increased age is a risk factor for EP. About 50% of EPs were treated without surgery or methotrexate. Increased use of treatment algorithms such as M4 will lead to increased use of expectant management in EP cases. Further research is needed.

## References

[1] Cantwell R (2011). Saving Mothers’ Lives: Reviewing maternal deaths to make motherhood safer: 2006–2008. The Eighth Report of the Confidential Enquiries into Maternal Deaths in the United Kingdom. BJOG.

[2] Sowter MC, Farquhar CM (2004). Ectopic pregnancy: an update. Current Opinion in Obstetrics and Gynecology.

[3] Grimes DA (2006). Estimation of pregnancy-related mortality risk by pregnancy outcome, United States, 1991 to 1999. American Journal of Obstetrics & Gynecology.

[4] Control CFD, Prevention (1995). Ectopic pregnancy–United States, 1990–1992. MMWR. Morbidity and mortality weekly report.

[5] Washington AE, Katz P (1993). Ectopic pregnancy in the United States: economic consequences and payment source trends. Obstetrics and gynecology.

[6] Chow W-H, Daling JR, Cates JR W, Greenberg RS (1987). Epidemiology of ectopic pregnancy. Epidemiologic reviews.

[7] Van Den Eeden SK, Shan J, Bruce C, Glasser M (2005). Ectopic pregnancy rate and treatment utilization in a large managed care organization. Obstetrics & Gynecology.

[8] Trabert B, Holt VL, Yu O, Van Den Eeden SK, Scholes D (2011). Population-based ectopic pregnancy trends, 1993–2007. American journal of preventive medicine.

[9] Hoover KW, Tao G, Kent CK (2010). Trends in the diagnosis and treatment of ectopic pregnancy in the United States. Obstetrics & Gynecology.

[10] Coste J (1994). Incidence of ectopic pregnancy. First results of a population-based register in France. Human Reproduction.

[11] Rajkhowa M (2000). Trends in the incidence of ectopic pregnancy in England and Wales from 1966 to 1996. BJOG: an international journal of obstetrics and gynaecology.

[12] Yuk JS, Kim YJ, Hur JY, Shin JH (2013). Association between socioeconomic status and ectopic pregnancy rate in the Republic of Korea. International Journal of Gynecology & Obstetrics.

[13] Kirk E, Bottomley C, Bourne T (2013). Diagnosing ectopic pregnancy and current concepts in the management of pregnancy of unknown location. Human reproduction update.

[14] van Mello, N. *et al*. Methotrexate or expectant management in women with an ectopic pregnancy or pregnancy of unknown location and low serum hCG concentrations? *A randomized comparison* (2013). 10.1093/humrep/des373 23081873

[15] Van Mello NM (2012). Ectopic pregnancy: how the diagnostic and therapeutic management has changed. Fertility and sterility.

[16] Mol F, Mol B, Ankum W, Van der Veen F, Hajenius P (2008). Current evidence on surgery, systemic methotrexate and expectant management in the treatment of tubal ectopic pregnancy: a systematic review and meta-analysis. Human reproduction update.

[17] Kim, L., Kim, J.-A. & Kim, S. A guide for the utilization of health insurance review and assessment service national patient samples. *Epidemiology and health* **36** (2014). 10.4178/epih/e2014008 PMC4151963 25078381

[18] Shalev E, Peleg D, Tsabari A, Romano S, Bustan M (1995). Spontaneous resolution of ectopic tubal pregnancy: natural history. Fertility and sterility.

[19] Berry J, Davey M, Hon M-S, Behrens R (2016). A 5-year experience of the changing management of ectopic pregnancy. Journal of Obstetrics and Gynaecology.

[20] Condous G (2007). Prediction of ectopic pregnancy in women with a pregnancy of unknown location. Ultrasound in obstetrics & gynecology.

[21] Choe, S. H. A Korean Doctors’ Group Wants to Halt Abortions. The New York Times. 10 Jan 2010. Available from: http://query.nytimes.com/gst/fullpage.html?res=9C02EFD7123EF933A25752C0A9669D8B63. Cited 10 Jan 2010.

[22] Weström L, Joesoef R, Reynolds G, Hagdu A, Thompson SE (1992). Pelvic inflammatory disease and fertility. A cohort study of 1,844 women with laparoscopically verified disease and 657 control women with normal laparoscopic results. Sexually transmitted diseases.

[23] Sunderam S (2015). Assisted Reproductive Technology Surveillance - United States, 2013. Morbidity and mortality weekly report. Surveillance summaries (Washington, D.C.: 2002).

[24] Sohn K (2017). Parents are rapidly getting older in South Korea. Human Fertility.

